# First person – Wedad Fallatah and Tara Smith

**DOI:** 10.1242/dmm.044032

**Published:** 2020-01-24

## Abstract

First Person is a series of interviews with the first authors of a selection of papers published in Disease Models & Mechanisms, helping early-career researchers promote themselves alongside their papers. Wedad Fallatah and Tara Smith are co-first authors on ‘Oral administration of a synthetic vinyl-ether plasmalogen normalizes open field activity in a mouse model of rhizomelic chondrodysplasia punctata’, published in DMM. Wedad is a PhD student in the lab of Dr Nancy Braverman at the Research Institute of the McGill University Health Center and McGill University, Montreal, QC, Canada, investigating mouse models to characterize and establish preclinical therapeutic interventions for rhizomelic chondrodysplasia punctate (RCDP). Tara is Vice President of Therapeutics in the lab of Dr Shawn Ritchie at Med-Life Discoveries LP, Saskatoon, SK, Canada. Her research focuses on evaluating plasmalogen precursors as potential therapeutic agents for the treatment of peroxisomal disorders such as RCDP, as well as neurodegenerative diseases of aging including Alzheimer's disease, Parkinson's disease and multiple sclerosis.


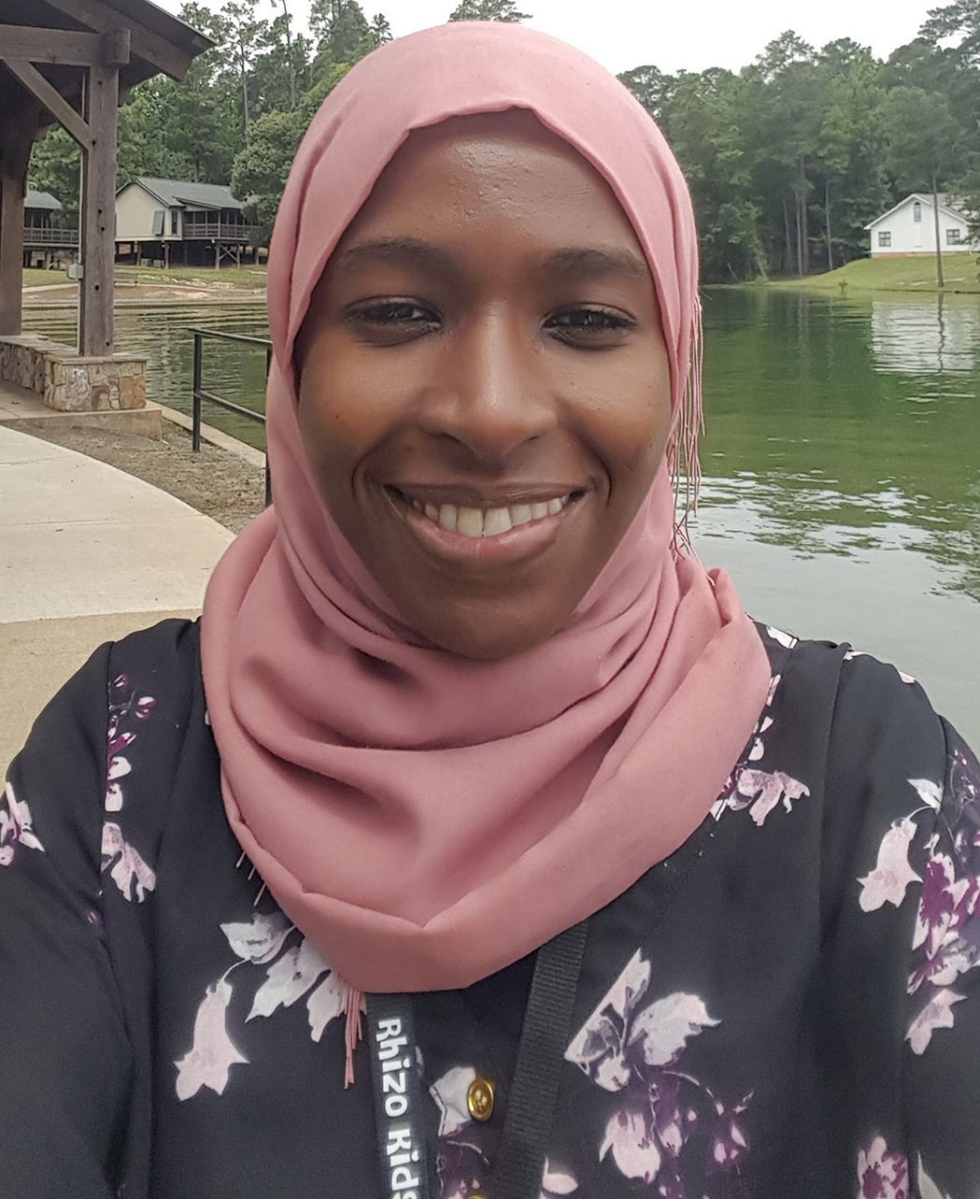


**Wedad Fallatah**


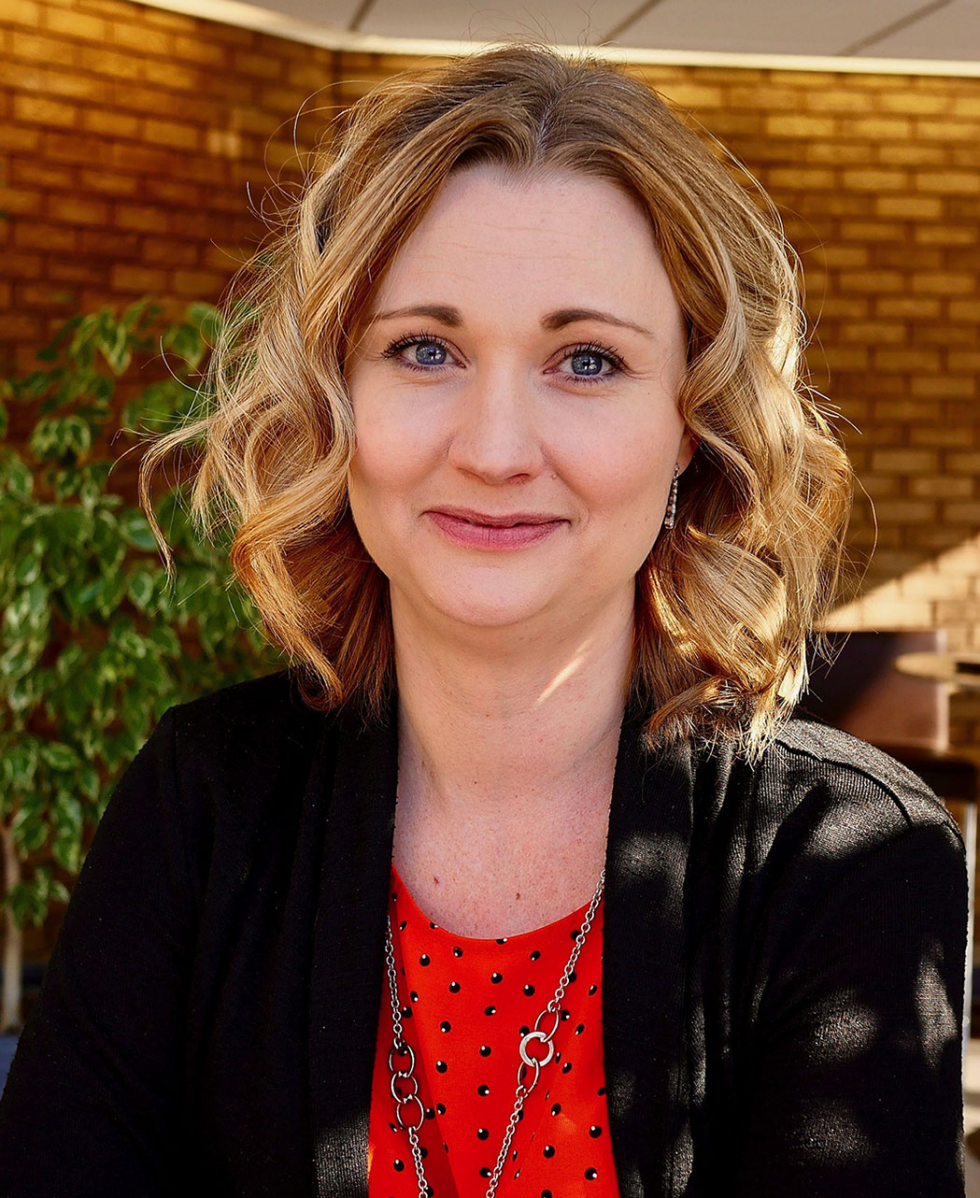


**Tara Smith**

**How would you explain the main findings of your paper to non-scientific family and friends?**

WF & TS: RCDP is a rare genetic disorder, caused by recessive mutations in genes involved in the biosynthesis of plasmalogens. Plasmalogens are a family of cell membrane phosphopholipids that contain a unique vinyl-ether bond, which imparts a crucial conformational change in the lipid. This change subsequently results in structural changes to cell membranes that impact a variety of membrane functions, including the ability to undergo vesicular fusion, a process required for cells to talk to one another. Patients with RCDP have severely reduced plasmalogen levels, resulting in a wide range of disease manifestations, and characteristically present with congenital cataract, skeletal defects and profound growth and developmental delays. Our study used a novel mouse model of RCDP to evaluate the feasibility of orally administering a new type of plasmalogen replacement. PPI-1040 contains the necessary vinyl-ether bond, eliminating any requirement by the body to convert a precursor into an intact plasmalogen.

“[…] augmentation in circulating plasmalogen levels correlated with a normalization of activity levels in mice, representing the first report of a behavioral effect following vinyl-ether plasmalogen treatment.”

**What are the potential implications of these results for your field of research?**

WF & TS: This is the first time that a synthetic vinyl-ether-containing plasmalogen has been shown to be orally bioavailable. The data show that the vinyl-ether bond remains intact following absorption in the gastrointestinal tract, and effectively augments a series of plasmalogen species in plasma and some tissues of the RCDP mouse model. This augmentation in circulating plasmalogen levels correlated with a normalization of activity levels in mice, representing the first report of a behavioral effect following vinyl-ether plasmalogen treatment. The real impact of these studies is that they validate PPI-1040 as a novel approach to improve plasmalogen levels in deficient animals and hopefully in the future that will translate into a new therapeutic strategy for RCDP patients.

**What are the main advantages and drawbacks of the model system you have used as it relates to the disease you are investigating?**

WF & TS: The RCDP mouse model used in this paper mirrors the biochemical manifestations of human RCDP, with reduced plasmalogen levels. This allowed for evaluation of plasmalogen augmentation following treatment with ether and vinyl-ether precursors. Overall though, the model does not reflect the severity of the phenotype most commonly observed in classic RCDP patients, limiting the ability to look for clinically meaningful changes in these mice. Interestingly, the mice do exhibit a hyperactive phenotype, which matches what has been observed in mildly affected RCDP patients. This hyperactivity allowed for the evaluation of the behavioral effect of treatment. The observed normalization of activity levels following PPI-1040 treatment is the most compelling data to date on the impact of plasmalogen augmentation *in vivo*.

**Figure:**
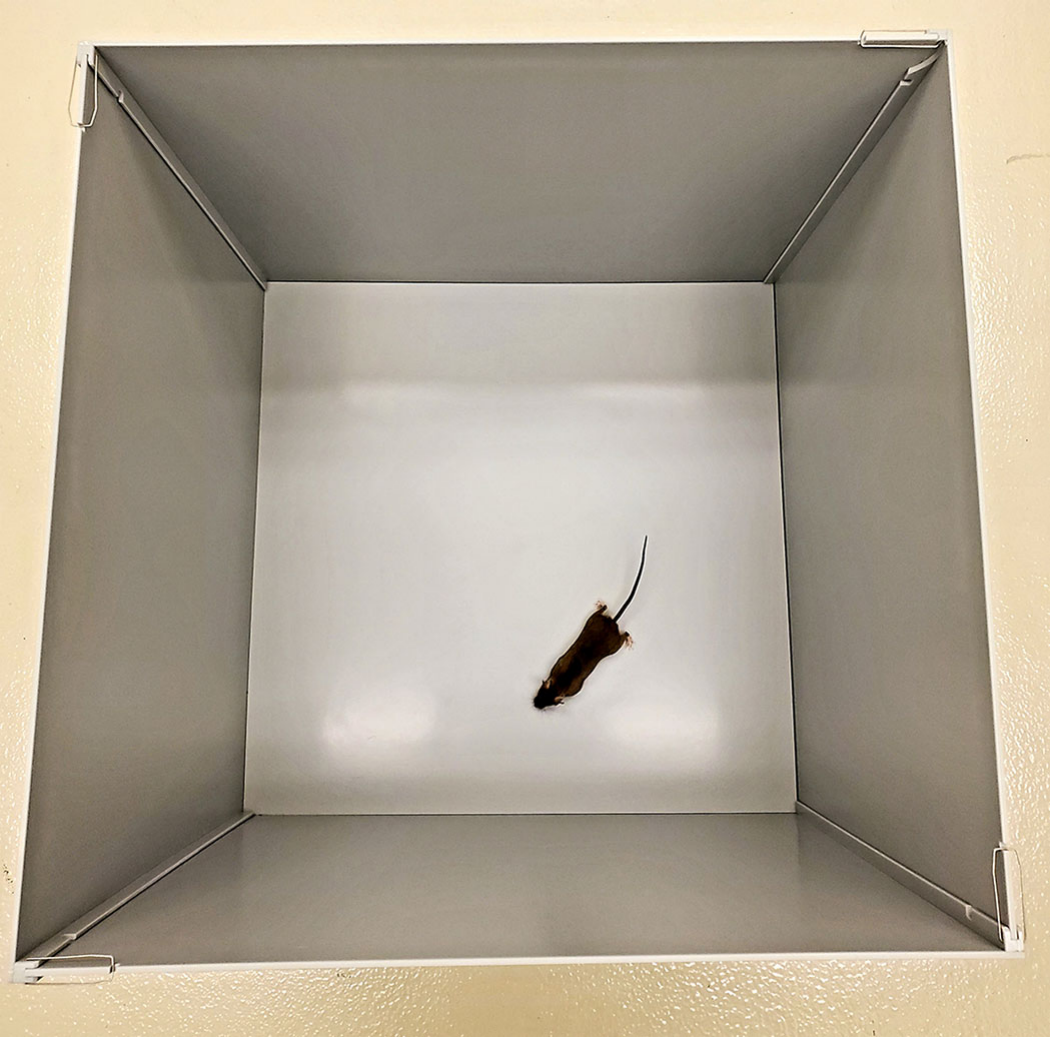
**The open field test that was used to evaluate the activity levels of the RCDP mouse model before and after plasmalogen treatment.**

**What has surprised you the most while conducting your research?**

WF & TS: The ability of PPI-1040 treatment to improve the hyperactive phenotype of the mice within such a short treatment period was surprising. This observation supports the idea that augmenting plasmalogen levels in deficient individuals can result in observable changes within a relatively short time period, suggesting that clinical evaluation of this approach may be warranted.

**Describe what you think is the most significant challenge impacting your research at this time and how will this be addressed over the next 10 years?**

WF: I think one of the most significant challenges in the treatment of rare genetic diseases like RCDP is finding the effective clinical endpoints that could be used to evaluate the treatment response in clinical settings. The challenge of finding measureable clinical endpoints is mainly due to limited knowledge of the natural history and pathophysiology of the disease, in addition to a relatively small number of patients. In addition, RCDP is a severe disease affecting multiple organs, which might require multiple approaches to target different organs and prenatal intervention to prevent the skeletal dysplasia. Within the next 10 years I think some of these challenges will be resolved, as the RCDP scientific and family community has already established a natural history study of RCDP that will help us to define valuable clinical endpoints and will improve our understanding of disease progression.

TS: While there is an increasing awareness of the importance of plasmalogens for proper cellular function, there remain a lot of unanswered questions on what functional and mechanistic effects would be expected with a plasmalogen precursor treatment. Over the next 10 years, I believe research will address these questions and clearly define the clinical role plasmalogen augmentation can play in the treatment of a variety of human diseases including RCDP.

**What changes do you think could improve the professional lives of early-career scientists?**

WF: I think early-career scientists should be open in exploring the wide range of career opportunities beyond academia, particularly in the field of translational research. While many scientists will follow the traditional path in finding jobs in university settings, there are a variety of options in industry and healthcare sectors that could also be beneficial for young scientists. I think organizing career-interest groups, workshops and internship programs will help young scientists to consider all available career paths following graduate study.

TS: I think there needs to be an increase in the level of mentorship for early-career scientists. So many aspects of a successful research program take place away from the lab and are not taught to PhD and postdoctoral students. I also think that scientists that have exposure to both the academic and industry sides of research are better situated to perform research that can be translated from the bench to the clinic.

**What's next for you?**

WF: I am currently working on clinical research to characterize the mild (non-classic) phenotype of RCDP in human patients to better understand disease progression in this specific group. My short-term goal is to finalize my PhD thesis and after that I will be looking for postdoctoral fellowship positions in the field of biochemical genetics and therapies for rare diseases.

TS: I remain committed to translating these exciting preclinical findings for PPI-1040 into a full-scale clinical evaluation of the drug in patients with RCDP. This will require completing extensive safety testing in animals and healthy adults before evaluating efficacy in RCDP patients. Longer term, my goal is to advance plasmalogen precursors toward the clinic for diseases of aging such as Alzheimer's disease.
